# Synovial fluid adipokines are associated with clinical severity in knee osteoarthritis: a cross-sectional study in female patients with joint effusion

**DOI:** 10.1186/s13075-016-1103-1

**Published:** 2016-09-15

**Authors:** Joan Calvet, Cristóbal Orellana, Jordi Gratacós, Antoni Berenguer-Llergo, Assumpta Caixàs, Juan José Chillarón, Juan Pedro-Botet, María García-Manrique, Noemí Navarro, Marta Larrosa

**Affiliations:** 1 Rheumatology Department, Parc Tauli Sabadell University Hospital, Institute for Research and Innovation Parc Tauli (I3PT), Universitat Autónoma de Barcelona (UAB), 08208 Sabadell, Spain; 2 Departament de Medicina, Universitat Autónoma de Barcelona (UAB), 08003 Barcelona, Spain; 3 Biostatistics and Bioinformatics Unit, Institute for Research in Biomedicine Barcelona (IRB Barcelona), 08028 Barcelona, Spain; 4 Endocrinology and Nutrition Department, Parc Tauli Sabadell University Hospital, Institute for Research and Innovation Parc Tauli (I3PT), Universitat Autónoma de Barcelona (UAB), 08208 Sabadell, Spain; 5 Endocrinology and Nutrition Department, Hospital del Mar, 08003 Barcelona, Spain

**Keywords:** Knee osteoarthritis, Adipokines, Inflammation, Synovial fluid, Clinical severity

## Abstract

**Background:**

Adipokines are related to knee osteoarthritis, but their exact role is not well known. The aim of this study was to evaluate the association between adipokines in synovial fluid and clinical severity in patients with knee osteoarthritis with joint effusion.

**Methods:**

Cross-sectional study with systematic inclusion of female patients with symptomatic primary knee osteoarthritis with ultrasound-confirmed joint effusion. Age, physical exercise, knee osteoarthritis symptoms duration, classical cardiovascular risk factors and different anthropometric measurements were collected. Metabolic syndrome was defined in accordance to National Cholesterol Education Program-Adult Treatment Panel III. Radiographic severity was evaluated according to Kellgren-Lawrence scale and Lequesne index was used to assess clinical severity. Seven adipokines (leptin, adiponectin, resistin, visfatin, osteopontin, omentin and chemerin) and three inflammatory markers (tumor necrosis factor α, interleukin 6 and high sensitivity C-reactive protein) were measured by enzyme-linked immunosorbent assay in synovial fluid.

**Results:**

Kellgren-Lawrence grade, physical exercise, all anthropometric measurements (especially waist circumference), tumor necrosis factor α, and high levels of leptin, resistin, and ostepontin were related to knee osteoarthritis severity. After adjustment for clinical confounders (age, symptom duration, and radiology), anthropometric measurements, inflammatory markers, and all evaluated adipokines, there were independent associations with clinical severity for resistin (directly associated) and visfatin (inversely associated). No other adipokines or inflammatory markers were independently associated with Lequesne index. The association of radiological parameters, physical exercise, and waist circumference with Lequesne index remained after adjustment.

**Conclusions:**

Resistin was directly associated, and visfatin was inversely associated, with clinical severity in female patients with knee osteoarthritis with joint effusion. These associations were more important after adjustment for confounders, especially when all adipokines were evaluated.

**Electronic supplementary material:**

The online version of this article (doi:10.1186/s13075-016-1103-1) contains supplementary material, which is available to authorized users.

## Background

Osteoarthritis (OA) is the most prevalent articular disease, and the most common cause of disability in developed and developing countries [1], with knee involvement being the most prevalent and disabling condition [2]. The prevalence of knee osteoarthritis (KOA) increases with age, and, given the aging of population, an even greater impact is expected in the future [3]. The etiology of OA is multifactorial, classically related to mechanical factors, trauma, or overload [2]. Genetic and, more recently, anthropometric, metabolic, and local inflammatory factors have been implicated in the pathophysiology of OA [4]. Different epidemiologic studies have shown a high prevalence of cardiovascular risk factors, such as hypertension, dyslipidemia, diabetes, obesity, and metabolic syndrome (MetS), in patients with KOA [5, 6]. MetS and its individual components are related to severity as measured by pain, disability, and radiography [7]. More recently, some authors have suggested that OA and MetS share a similar biochemical and inflammatory profile, which could explain associations between them and their influence in the severity or progression of the disease [8]. Obesity is, together with age, the most important risk factor related to KOA [9, 10]. In this respect, adipokines such as leptin, adiponectin, resistin, visfatin, and osteopontin, both in plasma and in synovial fluid, have been associated with the frequency and severity of KOA, usually measured by radiographic damage. However, studies of adipokines in synovial fluid were performed in patients with advanced disease undergoing prosthetic surgery and were not focused on severity or on the inflammatory profile of KOA [11–17].

Markers of inflammation in synovial fluid, such as interleukin (IL)-6, tumor necrosis factor α (TNF-α), or high-sensitivity C-reactive protein (hs-CRP), have been associated with severity in KOA in recent studies [18, 19]. In fact, a significant percentage of patients with KOA present with joint effusion, which could be considered as a marker of local inflammation. Patients with KOA with persisting joint effusion could be a particular subset of KOA with special characteristics compared with patients without effusion. These patients make up a group of patients of particular interest in investigations of metabolic inflammation in KOA.

The exact role of adipokines in KOA is not well known, but they may play a significant role indirectly via their link to obesity and directly on OA pathways in many patients. Researchers in different studies have evaluated the relationship between adipokines individually or controlled by some metabolic and other cytokines and clinical severity, but they did not assess the possible interactions when a significant number of adipokines were evaluated or their relationship to local inflammation. The main aim of this study was to evaluate the association of synovial adipokines with clinical severity in patients with KOA and persistent joint effusion. For this purpose, the effect on clinical severity and the biological interactions of adipokines previously studied in KOA were investigated.

## Methods

### Patients and study design

We conducted a cross-sectional study with systematic inclusion of female patients with symptomatic primary KOA according to American College of Rheumatology criteria [20] who visited our hospital for a monographic OA consultation. The women were aged 50–85 years and had evidence of significant joint effusion based on physical examination and confirmed by ultrasound (≥4 mm on midline suprapatellar line). Symptomatic OA was defined as pain intensity rated as ≥4 on a 10-cm visual analogue scale despite the use of prescribed analgesic drugs for at least 3 months. Only patients reporting persisting knee effusion or with documented effusion in several consultations were entered into the study. Patients with secondary OA were excluded, such as those with a history of trauma, meniscal injury, inflammatory rheumatic or septic conditions, previous knee surgery, any condition that could interfere with pain perception, systemic glucocorticoid intake in the last 6 months, or intra-articular glucocorticoid or hyaluronic acid injection in the last 3 or 6 months, respectively. The recruitment period was October 2013 to June 2015. We included only female patients to homogenize the sample, as there are differences between men and women related to pain perception, anthropometric measures, and fat content and distribution that might influence the adipokine profile [21–23]. This study was approved by the local ethics committee at the Parc Tauli Sabadell University Hospital. All patients included were verbally informed about the study and signed informed consent forms.

### Assessments

Information on the following variables was collected: age, physical exercise (never, occasional [less than 150 minutes per week], or regular), tobacco exposure, and KOA symptom duration. Each participant’s medical history, specifically regarding the presence of cardiovascular risk factors, was recorded. A diagnosis of hypertension, dyslipidemia, or diabetes was established if already diagnosed or if the participant was receiving treatment for any of these conditions. Anthropometric measurements included weight (kg), height (cm), body mass index (BMI) (kg/m^2^), waist circumference (WC) (cm), hip circumference (cm), waist-to-hip ratio (WHR), and percentage of body fat measured using a bioimpedance analyzer (BC-418 MA; Tanita, Arlington Heights, IL, USA) according to a standard protocol. Obesity was defined as a BMI ≥30 kg/m^2^. In accordance with the modified criteria of the National Cholesterol Education Program Adult Treatment Panel III, MetS was defined as having three or more of the following conditions: fasting plasma glucose ≥100 mg/dl or treatment with glucose-lowering drugs, arterial blood pressure ≥130/85 mmHg or antihypertensive medication, fasting plasma triglycerides ≥150 mg/dl (1.7 mmol/L) or drug treatment for hypertriglyceridemia, high-density lipoprotein (HDL) cholesterol <50 mg/dl (1.28 mmol/L) or drug therapy to raise HDL cholesterol concentration, and WC ≥88 cm [24]. Radiographic severity was evaluated by anteroposterior knee x-ray examination with the patient in standing position performed in the last 18 months and graded according to the Kellgren-Lawrence (KL) scale (grades 1–4). Time between radiologic evaluation and visit was considered for adjustment in the statistical analysis. Two rheumatologists (JC, CO) evaluated x-rays independently. The Lequesne algofunctional index, a simple and validated questionnaire for pain and disability in KOA with scores ranging from 0 (best) to 24 (worst), was used to assess clinical severity.

Joint aspiration was performed during the visit and at the same time of the day for proper evaluation of synovial adipokines. Synovial fluid was analyzed to ensure noninflammatory fluid (joint cell count <2500 cells) and absence of microcrystals. Synovial samples were stored at −80 °C. Seven adipokines and three inflammatory markers were measured by enzyme-linked immunosorbent assay (ELISA) in accordance with the manufacturers’ recommendations for synovial fluid dilutions: leptin (Biocompare, South San Francisco, CA, USA), adiponectin (eBioscience, San Diego, CA, USA), resistin (RayBiotech, Norcross, GA, USA), osteopontin (eBioscience), visfatin (Phoenix Pharmaceuticals, Burlingame, CA, USA), omentin (CUSABIO, Wuhan, China), chemerin (Elabscience, Bethesda, MD, USA), hs-CRP (DRG Diagnostics, Marburg, Germany), and IL-6 and TNF-α (Milliplex HCYTOMAG-60 K-03; Merck Millipore, Billerica, MA, USA) (see Additional file 1 for detailed description). For technical reasons related to ELISA technology (configuration of the plates used), none of these markers could be assessed at the same time for all patients. In order to control for technical variability, the rounds of measurement were considered as an adjustment factor in the statistical analyses.

### Statistical methods

Clinical data and laboratory parameters and their association with Lequesne index score were summarized using nonparametric methods. Medians, interquartile ranges, and Spearman’s correlations (*r*) were used for continuous measures, while frequencies and Mann-Whitney or Kruskal-Wallis tests were applied to categorical variables. For estimation of adjusted effects, linear models were fitted with suitable transformation of explanatory variables when necessary in order to fit the model assumptions. The covariates included in the multivariate analyses were age at recruitment, KL grade, time from visit to date of radiologic assessment, and KOA symptom duration. Using this model as a starting point, a stepwise algorithm was used sequentially with the rest of the potential confounders in two stages: first, only anthropometric and metabolic parameters were evaluated; second, inflammatory markers were considered in the resulting model, then each adipokine was added one at a time to the resulting model to assess its association with KOA severity (Table 3, “Adjusted effects” column). Finally, all adipokines were added to the model to assess their association with the Lequesne index after controlling for the anthropometric, metabolic, and inflammatory markers found to be informative in the previous steps, as well as for the rest of the adipokines (“Multivariate model” column in Table 3). For interpretation purposes, the partial correlation coefficient (PCC) was used as a measure of association for continuous variables. Associations for adipokines and inflammatory markers were adjusted by measurement round in all cases. Owing to high collinearity observed between adiponectin and omentin (PCC 0.792), only one of them at a time was included in a linear model. When selection of confounders was needed, a stepwise algorithm was carried out using Akaike’s information criterion for model selection. Associations were assessed in the linear models using the corresponding F and Wald tests. Tests were performed at the 5 % significance level. All statistical analyses were conducted using R software (see Additional file 2 for detailed description).

## Results

One hundred fifteen women were included (Table 1). The percentages of obesity and dyslipidemia were 57.4 % and 47.8 %, respectively. The prevalence of MetS was 40.9 %. The median BMI, WC, and WHR were in the obesity range (30.5 kg/m^2^, 100.5 cm, and 0.91, respectively). The median Lequesne index score was 14. The predominant KL grades were 2 and 3 (41.7 % each), and only 3.5 % of our sample were classified in KL grade 4. Because of batch measurement adjustments, we could not consider reference values or cutoff points for inflammatory markers or adipokines, but adjusted values are shown in Table 1.

**Table 1:** Demographic variables, cardiovascular risk factors, radiographic and clinical severity, anthropometric measurements, inflammatory markers, and adipokines levels in synovial fluid

Variables	Category		Median (IQR) or n (%)
Age, years			68.8 (11.1)
KOA symptoms duration, months			50.0 (73.0)
Tobacco exposure	Yes		8 (7.0 %)
Physical exercise	Never		53 (46.1 %)
	Occasional		28 (24.3 %)
	Regular		34 (29.6 %)
Cardiovascular risk factors	DM		12 (10.4 %)
	DL		55 (47.8 %)
	Obesity		65 (56.5 %)
	HT		63 (54.8 %)
	Crit MetS	0	10 (8.7 %)
		1	28 (24.3 %)
		2	31 (27.0 %)
		3	32 (27.8 %)
		4	14 (12.2 %)
	MetS		47 (40.9 %)
Anthropometric measurements	Body fat, %		41.8 (6.5)
	BMI, kg/m^2^		30.5 (6.4)
	Weight, kg		72.2 (13.5)
	WC, cm		100.5 (14.5)
	HC, cm		107.0 (14.0)
	WHR		0.91 (0.09)
Radiographic severity	KL grade	1	15 (13.1 %)
		2	48 (41.7 %)
		3	48 (41.7 %)
		4	4 (3.5 %)
Clinical severity	Lequesne index		14.0 (5.0)
Inflammatory markers in SF^a^	IL-6, pg/ml		106.0 (302.6)
	TNF-α, pg/ml		10.2 (8.0)
	hs-CRP, mg/ml		0.91 (0.76)
Adipokines in SF^a^	Leptin, pg/ml		42079.4 (29566.0)
	Adiponectin, ng/ml		1734.8 (1352.5)
	Resistin, pg/ml		2225.7 (2205.8)
	Visfatin, ng/ml		1.5 (1.2)
	Osteopontin, ng/ml		57.7 (83.2)
	Omentin, pg/ml		3396.0 (3550.4)
	Chemerin, ng/ml		102.7 (82.5)

*Abbreviations: DM* Diabetes mellitus, *DL* Dyslipidemia, *HT* Hypertension, *Crit MetS* Number of individual criteria for metabolic syndrome, *MetS* Metabolic syndrome, *KL* Kellgren-Lawrence scale, *BMI* Body mass index, *WC* Waist circumference, *HP* Hip circumference, *WHR* Waist-to-hip ratio. *IL-6* Interleukin 6, *SF* Synovial fluid, *TNF-α* Tumor necrosis factor-α, *hs-CRP* High-sensitivity C-reactive protein

Medians and interquartile ranges (IQR) were used to describe continuous variables; categorical data were summarized using absolute frequencies (n) and percentages (%)

^a^Levels of inflammatory markers and adipokines in synovial fluid were adjusted by measure round

Table 2 displays associations between Lequesne index; demographic, radiographic, and cardiovascular risk factors; and anthropometric measurements. Intensity of physical exercise showed a significant inverse association with clinical severity, and more severe KL grade was significantly associated with Lequesne index. All anthropometric parameters achieved significance, with WC being the one showing the strongest association (*r* = 0.404, *p* < 0.0001). It is worth noting that all these anthropometric measures were highly intercorrelated (*r* values 0.630–0.883). Weak, nonsignificant trends for association were found for MetS, obesity, and hypertension with Lequesne index (*p* = 0.072, *p* = 0.071, and *p* = 0.059, respectively). Among all the inflammatory markers under study, only TNF-α showed a significant association with Lequesne index after controlling by measurement batch (PCC 0.273, *p* = 0.0057). No association with severity was found for age, KOA symptom duration, MetS factors, dyslipidemia, or diabetes mellitus.

**Table 2:** Associations between Lequesne index and demographic, radiographic, and cardiovascular risk factors; anthropometric measurements; and inflammatory markers

	Category	Median or correlation (95 % CI)	*p* Value
Age		0.118 (−0.078, 0.313)	0.2090
KOA symptom duration (months)		−0.008 (−0.178, 0.176)	0.9345
Tobacco exposure	No	14.0 (13.0–15.0)	0.0608
	Yes	12.5 (7.0–15.0)	
Physical exercise	Never	15.0 (13.0–16.0)	0.0401
	Occasional	14.0 (12.0–16.0)	
	Regular	13.0 (10.0–14.0)	
Radiographic severity (KL grade)	1	13.0 (9.0–15.0)	0.1721
	2	13.0 (12.0–15.0)	
	3–4^a^	14.0 (13.0–16.0)	
DM	Yes	13.5 (11.0–18.0)	0.7480
	No	14.0 (13.0–15.0)	
DL	Yes	14.0 (12.0, 16.0)	0.8551
	No	14.0 (13.0–15.0)	
Obesity	Yes	14.0 (13.0–16.0)	0.0705
	No	13.0 (12.0–15.0)	
HT	Yes	14.0 (13.0–16.0)	0.0589
	No	13.0 (12.0–14.0)	
MetS	Yes	15.0 (13.0–16.0)	0.0722
	No	13.0 (12.0–14.0)	
Crit MetS	0	12.5 (9.0–16.0)	0.1490
	1	14.0 (13.0–15.0)	
	2	13.0 (11.0–14.0)	
	3	15.5 (14.0–17.0)	
	4	12.5 (10.0–18.0)	
Percent body fat		0.243 (0.062–0.422)	0.0088
BMI		0.277 (0.097–0.435)	0.0027
Weight		0.249 (0.082–0.407)	0.0072
WC		0.404 (0.237–0.567)	<0.0001
HC		0.252 (0.078–0.412)	0.0065
WHR		0.221 (0.026–0.392)	0.0177
IL-6		0.146 (−0.045, 0.327)	0.1259
TNF-α		0.273 (0.078–0.448)	0.0057
hs-CRP		0.139 (−0.051, 0.320)	0.1429

*Definition of abbreviations: KL* Kellgren-Lawrence scale, *DM* Diabetes mellitus, *DL* Dyslipidemia, *HT* Hypertension, *Crit MetS* Number of individual criteria for metabolic syndrome, *MetS* Metabolic syndrome, *BMI* Body mass index, *WC* Waist circumference, *HP* Hip circumference, *WHR* Waist-to-hip ratio, *IL-6* Interleukin 6, *TNF-α* Tumor necrosis factor-α, *hs-CRP* High-sensitivity C-reactive protein

Correlations for continuous variables, medians for groups and their corresponding 95 % confidence (95 % CI) are shown. The partial correlation coefficient after adjustment by measure round was used to assess associations for IL-6, TNF-α, and hs-CRP. Spearman’s correlation coefficient is shown for the rest of the continuous measures

^a^Because only four patients were classified as KL grade 4, KL grades 3 and 4 were combined in one category

Among all the anthropometric measures evaluated in a multivariate model, WC was the most strongly associated with clinical severity, accounting for an estimated increase of 0.9 points in the Lequesne index for every 5-cm increase in WC before simultaneous evaluation of inflammatory markers and adipokines (PCC 0.338, *p* = 0.0003). Although the percentage of body fat was also selected as a confounder according to model selection criteria, it was not significantly associated to severity in the multivariate model.

Table 3 shows the association between the Lequesne index and each adipokine for three different settings: in a univariate fashion (estimates were adjusted only by measurement batch), adjusting by selected confounders (a different model was fitted for each compound), and controlling by selected confounders as well as the rest of the adipokines (a unique model was fitted to perform all estimations). This sequential analysis allowed us to evaluate the relationship between each adipokine and OA severity while assessing the effect of confounders in these associations. Variables selected for adjustment of the adipokines’ effects included age, KL grade, time from recruitment to date of radiologic assessment, KOA symptom duration, WC, percentage of body fat, physical exercise, TNF-α, and batch of ELISA measurements.

**Table 3:** Association between adipokines and Lequesne index in three different settings

	Univariate effects		Adjusted effects		Multivariate model	
	PCC (95 % CI)	*p* Value	PCC (95 % CI)	*p* Value	PCC (95 % CI)	*p* Value
Leptin	0.413 (0.238–0.561)	<0.0001	0.182 (−0.033, 0.382)	0.0891	0.192 (−0.036, 0.402)	0.0897
Adiponectin	0.035 (−0.155, 0.222)	0.7170	0.134 (−0.078, 0.334)	0.2027	0.212 (−0.016, 0.419)	0.0612
Resistin	0.324 (0.143–0.483)	0.0005	0.179 (−0.031, 0.375)	0.0875	0.265 (0.040–0.464)	0.0184
Visfatin	0.066 (−0.125, 0.252)	0.4915	−0.179 (−0.374, 0.032)	0.0886	−0.298 (−0.492, −0.077)	0.0075
Osteopontin	0.350 (0.172–0.505)	0.0002	0.245 (0.037–0.432)	0.0187	0.197 (−0.032, 0.406)	0.0824
Omentin	0.013 (−0.179, 0.204)	0.8925	0.173 (−0.040, 0.371)	0.1028	0.216^a^ (−0.012, 0.422)	0.0562^a^
Chemerin	0.071 (−0.124, 0.260)	0.4674	0.037 (−0.179, 0.249)	0.7327	0.012 (−0.215, 0.239)	0.9133

For univariate effects, adipokine associations were assessed separately, adjusting their effects by measurement batch only. For adjusted effects, estimation of adipokine effects was additionally adjusted by potential confounders: age, knee osteoarthritis symptom duration, Kellgren-Lawrence grade (divided into three categories: 1, 2, and 3 + 4 combined), time from recruitment to radiology, waist circumference, percentage of body fat, physical exercise, and tumor necrosis factor α. In the multivariate model, effects were simultaneously estimated using a single model that included previous confounders and all adipokines except omentin (due to high collinearity observed with adiponectin: partial correlation coefficient [PCC] 0.792)

^a^Multivariate association for omentin was assessed in an analogous model in which adiponectin was excluded. The PCC after adjustment by measure round was used to assess associations between Lequesne index and adipokines

**Fig. 1:**
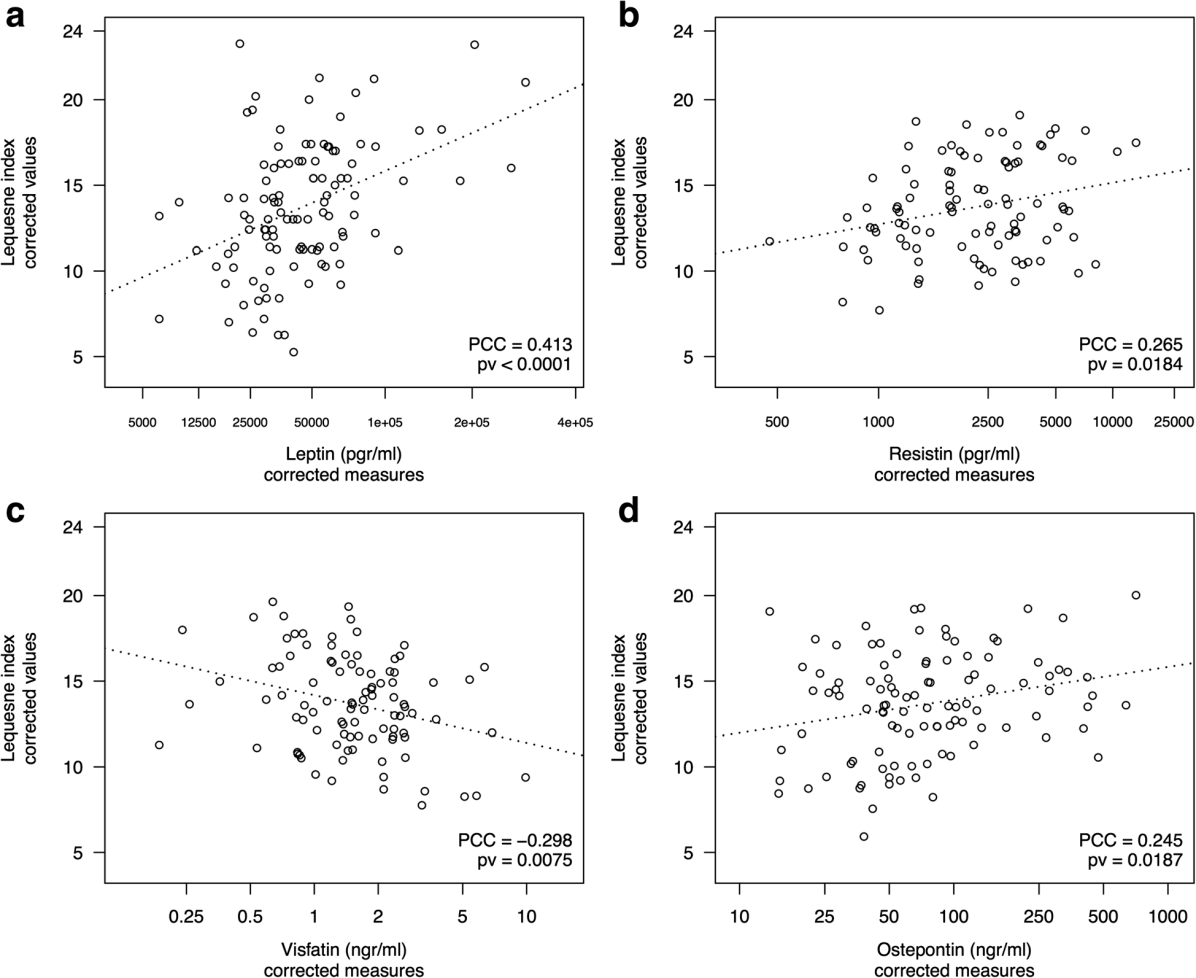
Scatterplots showing significant associations between adipokines and knee osteoarthritis severity: effect of leptin on Lequesne index score in the univariate setting (adjusted by round of measurement only (**a**), association for resistin (**b**), and visfatin (**c**) independently of clinical, anthropometric, metabolic, and inflammatory factors as well as the rest of the adipokines; association between Lequesne index and osteopontin after adjustment by clinical, anthropometric, metabolic, and inflammatory factors, but not the rest of the adipokines (**d**)). In all cases, values were corrected for confounders using the corresponding linear model. Adipokine values were drawn in the scale of the corresponding Taylor transformation. Labels along *x*-axes are shown in the original scale of the adipokines. *PCC* Partial correlation coefficient, *pv* Association *p* value according to *F*-test derived from the linear model

Regarding adipokines, leptin showed a significant strong association according to the univariate-like analysis (PCC 0.413, *p* < 0.0001) (Fig. 1a). However, significance was highly attenuated after adjustment by confounding variables, especially after inclusion of WC, as revealed by a detailed examination of the model (see Additional file 3: Table S2).

Similarly, in univariate analysis, resistin showed a highly significant correlation with Lequesne index (PCC 0.324, *p* = 0.0005) that was markedly reduced after adjustment by confounders. Nevertheless, resistin became the strongest positively associated adipokine when all of them were jointly evaluated (PCC 0.265, *p* = 0.0184) (Fig. 1b; see Additional file 3: Table S3).

**Fig. 2:**
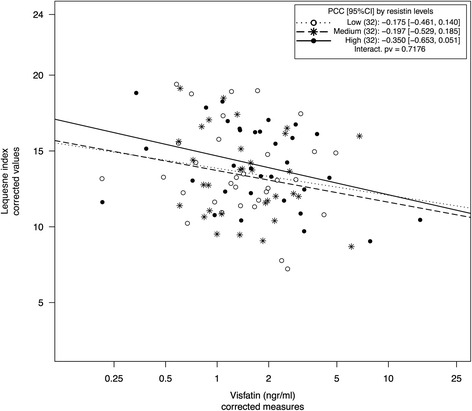
Scatterplot of visfatin vs Lequesne index after stratification by resistin levels. Values were corrected for confounders, except for resistin, using the complete model (multivariate model in Table 3). Levels of resistin were defined using tertiles after correction of their values by measurement round using the complete model. *PCC* Partial correlation coefficient, *95 % CI* PCC interval at 95 % confidence, *Interact. pv p* Value for the interaction between visfatin and resistin according to an *F*-test derived from the linear model

A nonsignificant association between visfatin and Lequesne index in the univariate analysis or after adjustment by selected confounders was found. Nevertheless, an inverse association became evident after inclusion of all adipokines in the model (PCC −0.298, *p* = 0.0075) (Fig. 1c). A detailed study of the model showed that this effect reached statistical significance after controlling for resistin levels (Fig. 2) (see Additional file 3: Table S4).

Osteopontin achieved a significant correlation with Lequesne index in the univariate and adjusted models (PCC 0.350, *p* = 0.0002, and PCC 0.245, *p* = 0.0187, respectively) (Fig. 1d). However, only a trend for association remained when it was evaluated in the complete model (PCC 0.197, *p* = 0.0824) (see Additional file 3: Table S5).

No relationship between adiponectin or omentin and Lequesne index was observed in the univariate or adjusted analyses, but a nonsignificant trend arose for both when the rest of the adipokines were included in the model (PCC 0.212, *p* = 0.0612, and PCC 0.216, *p* = 0.0562, respectively). Finally, chemerin showed no association with KOA clinical severity in any of the settings studied.

Regarding inflammatory markers, TNF-α showed a weak but statistically significant association with Lequesne index, which remained after adjustment by confounders (PCC 0.249, *p* = 0.0162). Nonetheless, there was no independent association between Lequesne index and TNF-α when controlled by the effect of adipokines. Specifically, a detailed study of the complete model revealed that TNF-α did not show an independent association for osteopontin and resistin (see Additional file 3: Table S1).

Last, the covariates that retained a significant association with Lequesne index after the inclusion of TNF-α and the adipokines in the complete model were physical exercise, with an estimated decrease of nearly 3 Lequesne index points among patients doing regular exercise versus sporadic or no exercise (*p* = 0.0016); WC, which showed an increase of 0.5 Lequesne index points for every 5-cm increment (PCC 0.230, *p* = 0.0419); and radiographic severity, with an average increase of 1 point in the Lequesne index for every change in KL grade (*p* = 0.0265) (see Additional file 3: Tables S6, S7, and S10).

## Discussion

In this study, we evaluated the relationship between clinical severity of KOA as measured by the Lequesne index and different adipokines and inflammatory markers in synovial fluid, anthropometric measurements, cardiovascular risk factors, and MetS in a cohort of women with KOA and persistent joint effusion. Our results show that, among all the adipokines measured, high levels of resistin, leptin, and osteopontin were related to greater clinical severity of KOA. Of these, resistin showed an effect independent from all adjusted variables; the association with leptin was attenuated by WC and was eliminated by TNF-α, while the effect of osteopontin did not remain once the rest of the adipokines were evaluated simultaneously. In addition, high levels of visfatin were found to be associated with lesser clinical severity for patients with similar levels of resistin. Regarding the rest of the clinical parameters, only WC among all anthropometric measurements was independently associated with Lequesne index score. Radiographic damage was also independently associated with clinical severity, while intensity of physical exercise was inversely related to Lequesne index score.

In our opinion, this cohort of patients is very homogeneous and has four distinctive features compared with previous studies. First, all patients in our sample had synovial effusion, which is usually considered an inflammatory feature, and this differentiates our study from others [25–27]. Second, our patients were very symptomatic in contrast to other series in which symptomatic status was not evaluated and only radiographic or diagnostic criteria were assessed [28–30]. Third, most patients were not in the final stage of the disease, with more than 50 % in KL grade 1 or 2, in contrast to other series where most patients had advanced disease or were undergoing prosthetic surgery. Fourth, all patients were women. As previous researchers have reported, there are differences regarding pain evaluation or anthropometric measures related to gender [31].

Resistin was the adipokine most clearly associated with the Lequesne index in our study. A previous study showed an association between Western Ontario and McMaster Universities Osteoarthritis Index (WOMAC) total score and synovial fluid resistin, but, in contrast to our study, it was conducted with patients undergoing arthroscopic lavage and was not focused on patients with joint effusion, and the relationship with Lequesne index was not evaluated [15]. Other authors have reported an association between resistin and IL-6 with severity scores of KOA [32], but patients were in a final stage of the disease, and the synovial fluid was collected during surgery. In contrast, other authors who have evaluated the role of synovial fluid IL-6 and TNF-α in patients with KOA stages similar to those of our sample found no relationship between IL-6 and WOMAC subscales of pain or disability [33]. Thus, the role of resistin in KOA clinical severity was not explained by the possible association with IL-6, which might be more important in more advanced disease [34].

Our analyses seem to unveil a probable protective effect of visfatin in KOA regarding clinical severity. Visfatin has been related to cartilage degradation [17], and synovial fluid levels of visfatin have been associated with hip pain, but not with knee pain [35]. In contrast to our study, in these studies all patients were classified in KL grade 3 or 4 and undergoing joint arthroplasty. In vitro studies had evaluated the relationship between visfatin and different pain mechanisms in OA, such as nerve growth factor stimuli and nicotinamide phosphoribosyltransferase enzyme activity [36, 37]. Nevertheless, to our knowledge, its association with OA severity has not been clinically evaluated. Interestingly, the association with visfatin becomes evident only when controlled by TNF-α and the rest of the adipokines in the analysis, especially resistin. We do not know the mechanism behind this effect, but this result suggests the existence of interrelationships among these compounds regarding their role in KOA clinical severity. Overall, these findings may provide new and promising hypotheses for exploration in future research into how inflammation is activated or blocked by visfatin. In this respect, it might be interesting to assess the association of synovial fluid visfatin levels with anti-inflammatory factors, such as IL-10 [38].

Previous studies have related synovial fluid osteopontin with KOA severity [16, 39], although the patients differed from those in our series because of synovial fluid presence and KOA stage. In our study, osteopontin showed a clear association with clinical KOA severity that decreased when adjusted by other adipokines, again suggesting the existence of biological or clinical interrelationships between adipokines. An interaction between osteopontin and resistin has been shown in vascular disease [40], and it is possible that synovial osteopontin could also be controlled by other adipokines in KOA or that they could share mechanisms of action.

A relationship between leptin and clinical severity in knee OA has already been reported [35, 41], but, to our knowledge, this is the first study where synovial leptin has been studied simultaneously with a significant number of adipokines. After proper control by WC, the association with leptin was highly attenuated and lost statistical significance when controlled by TNF-α. Interaction between leptin and BMI in knee OA has been described previously using serum samples and related to radiologic KOA, but clinical severity was not evaluated, and this study was not controlled by the presence of inflammatory features such as joint effusion [42]. Leptin and anthropometric measurements may share pathways influencing clinical severity in KOA, but our results could indicate that WC may have a more important association than leptin in patients with joint effusion.

Regarding resistin, visfatin, leptin, and osteopontin, it is important to highlight that when a model excluding anthropometric measures or inflammatory factors was applied (see Additional file 3: Tables S8 and S9), the association of clinical severity with resistin and visfatin persisted, suggesting that their effects were not completely explained by obesity or TNF-α. The association between Lequesne index and leptin was highly increased in the two models when the effect of leptin was not controlled by anthropometric or inflammatory variables, indicating that the pathway of leptin in clinical severity could be shared by TNF-α and obesity. The relationship of clinical severity and osteopontin was higher when not controlled by anthropometric measures, but it was attenuated when not controlled by TNF-α, indicating a possible common pathway between obesity and osteopontin [43].

A nonsignificant trend toward an association between adiponectin and omentin with Lequesne index was observed when other adipokines were considered. Adiponectin has been found to be associated with inflammatory synovial parameters, but not with clinical severity [12, 44]. Researchers in one previous study found that synovial fluid omentin was associated with less pain and disability as measured by WOMAC but that it was not controlled by other adipokines [45]. One important deduction from our results is that, in order to evaluate the effect of adipokines properly, as many of them as possible should be assessed together and controlled by anthropometric variables and inflammatory markers.

The association of Lequesne index with TNF-α remained significant after controlling for clinical, anthropometric, and metabolic factors. Nevertheless, this association did not appear to be independent from the effect of adipokines, especially ostepontin and resistin. In a previous study with a group of patients comparable to ours, investigators found a relationship between TNF-α, pain, and disability as measured using WOMAC subscales, but it was not controlled by other adipokines [33]. Further work might be required to unveil the role of TNF-α in clinical KOA severity and its relationship to adipokines [29].

Among all anthropometric measurements, WC was the most strongly associated with clinical severity. Different anthropometric measurements have been related to KOA prevalence in prior studies, and their relationship to Lequesne index has been described previously [30, 46, 47]. WC could represent a better measurement of visceral and abdominal fat, which is known to be related to low-grade systemic inflammation [9, 10], and could have more relevance in our group of patients with local inflammation.

Several studies have connected physical exercise with less pain and disability in KOA. It has been suggested that this effect could be related to better muscle strength [48]. In our study, physical exercise was associated with a better Lequesne index score.

Cross-sectional studies are not the best way to evaluate radiographic implication in pain, but disability may be evaluated. In our study, the relationship between radiologic and clinical severity was assessed in a group of female patients with KOA with effusion. We used the Lequesne index, in which disability has a relative weight greater than that of pain, and we found that radiographic severity was associated with Lequesne index, in accordance with previous studies [49], although they were not conducted with patients with KOA with synovial effusion.

Cardiovascular risk factors and MetS have been associated with the presence of OA [6, 7], and in some cases with higher pain levels, especially in patients with diabetes. Association between MetS and KOA was not evaluated in this study, but MetS and its individual components were very prevalent in our patients. No individual cardiovascular risk factor was related to Lequesne index, although hypertension and obesity showed a trend toward association. Neither MetS nor the addition of its individual components was significantly associated with clinical severity in our group of female patients with KOA on the basis of synovial fluid measurements. It is important to highlight that the association between MetS and OA may change depending on the diagnostic criteria used, so it would be important to standardize criteria for future studies [46, 50].

The main limitation of our study arises from its cross-sectional nature and therefore its inability to establish causality. Accordingly, conclusions can be drawn only in terms of associations. A selection bias toward greater disease severity could exist, as all patients were referred from primary care or other specialists to our rheumatology unit and were systematically included. These results warrant replication in other groups of patients with KOA, such as men or patients with lesser WC, BMI, or pain. In this study, information on OA at other sites, which could interfere with the evaluation of clinical severity, was not adequately collected to be analyzed. There may be technical concerns regarding measures in synovial fluid that are inherent to ELISA technology; nonnegligible effects associated with time of measurement were identified. For this reason, we corrected these measures by round in the statistical models to make values of adipokines and inflammatory markers totally comparable across samples. Although this correction resulted in reliable estimations of association for these measures, it was not possible to establish either their real range of variability or meaningful cutoffs that could be extrapolated to other datasets.

A remarkable strength of this work is that a highly homogeneous sample of patients with KOA was studied, which increased the statistical power to detect associations of a moderate magnitude. Another strength is the availability of patient information relevant to outcome, which makes this study singular among others, as it allowed for a simultaneous analysis of a high number of clinical, anthropometric, metabolic, and inflammatory factors and their relationship to up to seven different adipokines.

## Conclusions

To the best of our knowledge, this is the first study of the relationship between seven adipokines in synovial fluid and clinical severity of KOA controlled by three inflammatory markers in synovial fluid, anthropometric measurements, and metabolic factors. Resistin and visfatin were independently associated with the Lequesne index. Leptin and osteopontin were associated with clinical severity of KOA until all adipokines were evaluated together, pointing to potential biologic interrelationships among them. Therefore, simultaneous study of different adipokines should be recommended in future research. Further evaluation of different adipokines both in synovial fluid and in serum in future studies and in different populations, such as male patients, patients without synovial effusion, or patients with lower levels of pain are warranted.

## Additional files

Additional file 1: Detailed description for kit measurement. (DOCX 11 kb) Additional file 2: More detailed description of statistical methods. (DOCX 13 kb) Additional file 3: Supplementary tables. (DOCX 54 kb)

## Abbreviations

BMI: Body mass index
DL: Dyslipidemia
DM: Diabetes mellitus
HC: Hip circumference
HDL: High-density lipoprotein
hs-CRP: High-sensitivity C-reactive protein
HT: Hypertension
IL: Interleukin
KL: Kellgren-Lawrence
KOA: Knee osteoarthritis
MetS: Metabolic syndrome
OA: Osteoarthritis
PCC: Partial correlation coefficient
pv: *p* value
*r*: Spearman’s correlation coefficient
SF: Synovial fluid
TNF-α: Tumor necrosis factor α
WC: Waist circumference
WHR: Waist-to-hip ratio
WOMAC: Western Ontario and McMaster Universities Osteoarthritis Index
