# Supplementary material for: Synovial fluid adipokines are associated with clinical severity in knee osteoarthritis: a cross-sectional study in female patients with joint effusion
Source: Arthritis Res Ther. 2016 Sep 15;18:207. doi: 10.1186/s13075-016-1103-1 (PMC5024464; doi:10.1186/s13075-016-1103-1)
Supplement: Additional file 1: — Detailed description for kit measurement. (DOCX 11 kb) [file 13075_2016_1103_MOESM1_ESM.docx]

*Kits of measurements*

Seven adipokines were measured in synovial fluid: leptin, adiponectin, resistin, osteopontin, visfatin, omentin and chemerin by ELISA following manufacturer recommendations for synovial fluid dilutions. Kit characteristics used were the following: Leptin: Human Leptin ELISA Kit (Biocompare, California, USA). Dilution 1/100. Sensibility: < 8 pg/ml, detection rang: 62.5 - 10000 pg/L, Intra-assay: <6.3%, Inter-assay: <7.2%. Adiponectin: Adiponectin ELISA kit (eBioscience, California, USA). Dilution 1/1000. Sensibility: 0.01 ng/ml, detection rang: 0.78-50 mg/L, Coef. intra-assay: 4.2%, Coef. inter-assay: 3.1%. Resistin: Human Resistin ELISA Kit (Raybiotech, GA, USA). Dilution  1/100. Sensibility: 1.4pg/ml, detection rang: 1.4-400 pg/ml, Coef. intra-assay: <10%, Coef. inter-assay: <12%. Osteopontin: Osteopontin ELISA kit (eBioscience, California, USA). Dilution 1/100. Sensibility: 0.26 ng/ml, detection rang: 0.47-30 mg/L, Coef. intra-assay: 6.7%, Coef. inter-assay: 6.1%. Visfatin: Visfatin ELISA kit (Phoenix Pharmaceuticals, California, USA). Dilution: none. Sensibility: 2.21 ng/ml, detection rang: 0.1-1000 ng/ml, Coef. intra-assay: <10%, Coef. inter-assay: <15%. Omentin: Cusabio (China) ref. CSB-E09745h, Sensibility: 0.4 pg/ml, detection rang: 1.56 - 100 pg/ml, Coef. variance intra-assay: <8%, inter assay: <10%. Chemerin: Elabscience (China) ref. E-EL-H0698, Sensibility: 0.094ng/ml, detection rang: 0.156-10ng/ml, Coef. intra and inter assay: <10% . Three inflammatory markers were measured in synovial fluid: hs-CRP, IL6 and TNF-alpha. Kit characteristics used were the following: hs-PCR: EIA Kit (DRG Diagnostics, Germany).  Dilution 1/500. Sensibility: 0.1 mg/L. Detection Rang: 0.005 -0.1 mg/L, Coef. intra-assay: <10%, Coef. inter-assay: <5%. Luminex: HCYTOMAG-60K-03, (Merck Millipore) Dilution: direct. Sensibilitat: IL6: 0.9 pg/ml, TNF-alpha: 0.7 pg/ml, detection rang: 3.2 - 2000 pg/L, Coef. intra-assay:  IL6: 2%, TNF-alfa: 2.6%, Coef. inter-assay: IL6: 18.3%, TNF-alpha: 13%.
