# Supplementary material for: Synovial fluid adipokines are associated with clinical severity in knee osteoarthritis: a cross-sectional study in female patients with joint effusion
Source: Arthritis Res Ther. 2016 Sep 15;18:207. doi: 10.1186/s13075-016-1103-1 (PMC5024464; doi:10.1186/s13075-016-1103-1)
Supplement: Additional file 2: — More detailed description of statistical methods. (DOCX 13 kb) [file 13075_2016_1103_MOESM2_ESM.docx]

*Statistical methods*

Clinical information and laboratory parameters were summarized using medians and interquartilic ranges (continuous measures) and frequencies (categorical variables).

Descriptives of adipokines and inflammatory markers TNF-alpha, hs-CRP or IL6 were calculated after removing the effect of the measurement round, using a linear model which included batch as the only explanatory variable.

Univariate association with Lequesne score was assessed by means of non-parametric methods; for numeric variables, Spearman correlations (r) and their corresponding assympotic 95% confidence intervals and p-values were computed; association with group was assessed using a Mann-Whitney (when binary) or a Kruskal-Wallis (when more than 2 levels) test; median groups were estimated together with their corresponding 95% confidence intervals based on the binomial distribution. Due to the presence of only 4 patients in Kellgren-Lawrence scale grade 4, for assessment of association and for its inclusion as confouding factor, we divided the KL scale into three categories where grade 3 is a combination of grade 3 plus 4. Grade 1 and 2 are as in the definition of the KL scale. For assessment of association between Lequesne index and each of the adipokines or inflammatory markers, TNF-alpha, hs-CRP or IL6, a linear model was used in which the round of measurement was included as a covariate (see Table 2 for inflammatory markers and univariant effects in Table 3 for adipokines).

To estimate the influence of known and potential risk factors on OA severity while controlling by the rest of clinical and technical parameters, a multivariate linear model was fitted to the Lequesne score. The covariates included in the multivariate analyses were age at recruitment, KL, time from visit to date of radiologic assessment and time of evolution of the OA disease (symptoms duration). Using this model as starting point, a stepwise algorithm was carried out sequentially on the rest of potential confounders in two stages: firstly, only anthropometric and metabolic paremeters were evaluated for inclusion in the model; next, inflammatory markers were considered in the resulting model in order to assess their additional contribution as explanatory variables of the Lequesne score. Then, each adipokine was added at a time to the resulting model in order to assess its association with KOA severity controlling by the selected confounders (Table 3, Adjusted effects). Finally, all adipokines were added to the model in order to assess their association with the Lequesne index after controlling for the anthropometric, metabolic and inflammatory markers found to be informative in the previous steps, as well as for the rest of adipokines (Multivariate model in Table 3). Due to a high collinearity observed between adiponectin and omentin, only adiponectin was included in the final model as it showed to be the most informative; assessment of association for omentin in this setting was perfomed using an analogous model in which adiponectin was replaced by omentin. Batch of measurement was included as covariate whenever an adipokine or TNF-alpha, hs-CRP or IL6 were also present in the model. In all these analyses, Akaike’s Information Criteria (AIC) was used for model selection.

When a linear model was involved, Partial Correlation Coefficients (PCC) and the adjusted group means and regression coefficients provided by the models were used as measures of association for continuous variables. For each of these measures, intervals at 95% confidence were built. Associations in the linear models were assessed by means of the corresponding F and Wald tests. Threshold for statistical significance was set at 5%. When needed, the continuous explanatory variables were transformed using a proper Tukey's transformation in order to fulfill the assumptions of the linear model. Lambda parameters selected for transformations were 0 (i.e., logarithmic transformation: visfatin, ostepontin, omentin and IL6); 0.25 (leptin, TNF-alpha and KOA symptoms duration); 0.50 (hs-CRP); -0.25 (resistin); -0.50 (adiponectin, chemerin).

For visualization purposes, scatter plots for Lequesne index and adipokines were built. In order to show the correlation of the confounders considered independently, values of the Lequesne score and the adipokines were corrected by covariables using the corresponding linear model. For the association analysis of visfatin strafied by levels of resistin, intervals at 95% were built for PCC using bootstrap. Levels of resistin were defined using tertiles after correction of their values by measurement round using the final model. Statistical significance for the interaction between visfatin and resistin was assessed using a F-test derived from the linear model. All statistical analyses were conducted using R (R Core Team (2014)).

[R: A language and environment for statistical computing. R Foundation for Statistical Computing, Vienna, Austria. URL <http://www.R-project.org/>.)]
