# Supplementary material for: Synovial fluid adipokines are associated with clinical severity in knee osteoarthritis: a cross-sectional study in female patients with joint effusion
Source: Arthritis Res Ther. 2016 Sep 15;18:207. doi: 10.1186/s13075-016-1103-1 (PMC5024464; doi:10.1186/s13075-016-1103-1)
Supplement: Additional file 3: — Supplementary tables. (DOCX 54 kb) [file 13075_2016_1103_MOESM3_ESM.docx]

**Table S1**: partial correlation coefficients and their significance for TNF-alpha after sequential addition of selected confounders and the rest of adipokines.

| Variable added | PCC [95%CI] | p-value | PCC % Change |
| --- | --- | --- | --- |
| Univariate | 0.273 [0.078, 0.448] | 0.00569 | ***100.0%*** |
| KL | 0.272 [0.076, 0.448] | 0.00618 | 99.6% |
| Age | 0.259 [0.061, 0.438] | 0.00953 | 94.9% |
| KOA symptoms duration | 0.255 [0.055, 0.435] | 0.0112 | 93.4% |
| Time from radiology | 0.255 [0.054, 0.436] | 0.0118 | 93.4% |
| Physical exercise | 0.299 [0.099, 0.475] | 0.00328 | 109.5% |
| WC | 0.265 [0.061, 0.447] | 0.00991 | 97.1% |
| % Body Fat | 0.249 [0.043, 0.435] | 0.0162 | 91.2% |
| Osteopontin SF | 0.174 [-0.037, 0.370] | 0.0979 | 63.7% |
| Leptin SF | 0.145 [-0.073, 0.350] | 0.1797 | 53.1% |
| Visfatin SF | 0.209 [-0.008, 0.408] | 0.0535 | 76.6% |
| Resistin SF | 0.138 [-0.083, 0.346] | 0.2076 | 50.5% |
| Adiponectin SF | 0.128 [-0.094, 0.338] | 0.2462 | 46.9% |
| Chemerin SF | 0.131 [-0.099, 0.348] | 0.2497 | 48.0% |

**PCC**: Partial Correlation Coefficient; **95%**CI: intervals at 95% confidence; **PCC %Change:**change of partial correlation coefficient in percentage compared to the reference model (100%, cursive bold text). **KL:** Kellgren-Lawrence grade (divided into three categories 1, 2 and 3+4 combined), **WC:** Waist Circumference; **SF:** synovial fluid.

**Table S2**: partial correlation coefficients and their significance for leptin after sequential addition of selected confounders and the rest of adipokines.

| Variable added | PCC [95%CI] | p-value | PCC % Change |
| --- | --- | --- | --- |
| Univariate | 0.413 [0.238, 0.561] | 1.01e-05 | ***100.0%*** |
| KL | 0.419 [0.244, 0.567] | 7.8e-06 | 101.5% |
| Age | 0.408 [0.231, 0.559] | 1.57e-05 | 98.8% |
| KOA symptoms duration | 0.409 [0.232, 0.561] | 1.59e-05 | 99.0% |
| Time from radiology | 0.408 [0.230, 0.561] | 1.85e-05 | 98.8% |
| WC | 0.249 [0.053, 0.426] | 0.0116 | 60.3% |
| Physical exercise | 0.196 [-0.005, 0.381] | 0.0509 | 47.5% |
| % Body Fat | 0.231 [0.031, 0.413] | 0.0216 | 55.9% |
| TNF-alpha SF | 0.182 [-0.033, 0.382] | 0.0891 | 44.1% |
| Osteopontin SF | 0.167 [-0.051, 0.369] | 0.1226 | 40.4% |
| Visfatin SF | 0.183 [-0.036, 0.384] | 0.0923 | 44.3% |
| Resistin SF | 0.169 [-0.051, 0.373] | 0.1223 | 40.9% |
| Adiponectin SF | 0.167 [-0.055, 0.373] | 0.1297 | 40.4% |
| Chemerin SF | 0.192 [-0.036, 0.402] | 0.0897 | 46.5% |

**PCC**: Partial Correlation Coefficient; **95%**CI: intervals at 95% confidence; **PCC %Change:**change of partial correlation coefficient in percentage compared to the reference model (100%, cursive bold text). **KL:** Kellgren-Lawrence grade (divided into three categories 1, 2 and 3+4 combined), **WC:** Waist Circumference; **SF:** synovial fluid.

**Table S3**: partial correlation coefficients and their significance for resistin after sequential addition of selected confounders and the rest of adipokines.

| Variable added | PCC [95%CI] | p-value | PCC % Change |
| --- | --- | --- | --- |
| Univariate | 0.324 [0.143, 0.483] | 0.000501 | ***100.0%*** |
| KL | 0.347 [0.168, 0.503] | 0.000193 | 107.1% |
| Age | 0.332 [0.150, 0.491] | 0.000404 | 102.5% |
| KOA symptoms duration | 0.332 [0.150, 0.492] | 0.000424 | 102.5% |
| Time from radiology | 0.330 [0.147, 0.491] | 0.000485 | 101.9% |
| WC | 0.284 [0.096, 0.453] | 0.00302 | 87.7% |
| Physical exercise | 0.250 [0.057, 0.425] | 0.0101 | 77.2% |
| % Body Fat | 0.234 [0.039, 0.411] | 0.0168 | 72.2% |
| TNF-alpha SF | 0.179 [-0.031, 0.375] | 0.0875 | 55.2% |
| Osteopontin SF | 0.131 [-0.082, 0.332] | 0.2175 | 40.4% |
| Visfatin SF | 0.185 [-0.028, 0.382] | 0.0808 | 57.1% |
| Adiponectin SF | 0.227 [0.014, 0.419] | 0.0328 | 70.1% |
| Leptin SF | 0.277 [0.061, 0.468] | 0.0107 | 85.5% |
| Chemerin SF | 0.265 [0.040, 0.464] | 0.0184 | 81.8% |

**PCC**: Partial Correlation Coefficient; **95%**CI: intervals at 95% confidence; **PCC %Change:**change of partial correlation coefficient in percentage compared to the reference model (100%, cursive bold text). **KL:** Kellgren-Lawrence grade (divided into three categories 1, 2 and 3+4 combined), **WC:** Waist Circumference; **SF:** synovial fluid.

**Table S4**: partial correlation coefficients and their significance for visfatin after sequential addition of selected confounders and the rest of adipokines.

| Variable added | PCC [95%CI] | p-value | PCC % Change |
| --- | --- | --- | --- |
| Univariate | 0.066 [-0.125, 0.252] | 0.4915 | -36.9% |
| KL | 0.047 [-0.144, 0.235] | 0.6225 | -26.3% |
| Age | 0.021 [-0.171, 0.211] | 0.8303 | -11.7% |
| KOA symptoms duration | 0.023 [-0.170, 0.214] | 0.8136 | -12.8% |
| Time from radiology | 0.025 [-0.168, 0.217] | 0.7971 | -14.0% |
| WC | -0.032 [-0.224, 0.163] | 0.7442 | 17.9% |
| Physical exercise | -0.072 [-0.264, 0.125] | 0.4659 | 40.2% |
| % Body Fat | -0.081 [-0.273, 0.117] | 0.4137 | 45.3% |
| TNF-alpha SF | -0.179 [-0.374, 0.032] | 0.0886 | ***100.0%*** |
| Osteopontin SF | -0.176 [-0.373, 0.036] | 0.0954 | 98.3% |
| Resistin SF | -0.219 [-0.412, -0.007] | 0.0382 | 122.3% |
| Adiponectin SF | -0.263 [-0.451, -0.053] | 0.0128 | 146.9% |
| Leptin SF | -0.302 [-0.489, -0.088] | 0.00524 | 168.7% |
| Chemerin SF | -0.298 [-0.492, -0.077] | 0.00754 | 166.5% |

**PCC**: Partial Correlation Coefficient; **95%**CI: intervals at 95% confidence; **PCC %Change:**change of partial correlation coefficient in percentage compared to the reference model (100%, cursive bold text). **KL:** Kellgren-Lawrence grade (divided into three categories 1, 2 and 3+4 combined), **WC:** Waist Circumference; **SF:** synovial fluid.

**Table S5**: partial correlation coefficients and their significance for osteopontin after sequential addition of selected confounders and the rest of adipokines.

| Variable added | PCC [95%CI] | p-value | PCC % Change |
| --- | --- | --- | --- |
| Univariant | 0.350 [0.172, 0.505] | 0.000158 | ***100.0%*** |
| KL | 0.353 [0.174, 0.508] | 0.000147 | 100.9% |
| Time from radiology | 0.354 [0.175, 0.511] | 0.000146 | 101.1% |
| Age | 0.338 [0.157, 0.498] | 0.00032 | 96.6% |
| KOA symptoms duration | 0.337 [0.154, 0.497] | 0.000361 | 96.3% |
| WC | 0.295 [0.107, 0.462] | 0.00205 | 84.3% |
| Physical exercise | 0.303 [0.114, 0.470] | 0.0017 | 86.6% |
| % Body Fat | 0.285 [0.094, 0.456] | 0.00338 | 81.4% |
| TNF-alpha SF | 0.245 [0.037, 0.432] | 0.0187 | 70.0% |
| Leptin SF | 0.277 [0.066, 0.465] | 0.00931 | 79.1% |
| Visfatin SF | 0.276 [0.062, 0.465] | 0.0102 | 78.9% |
| Resistin SF | 0.241 [0.024, 0.436] | 0.0264 | 68.9% |
| Adiponectin SF | 0.185 [-0.036, 0.389] | 0.0918 | 52.9% |
| Chemerin SF | 0.197 [-0.032, 0.406] | 0.0824 | 56.3% |

**PCC**: Partial Correlation Coefficient; **95%**CI: intervals at 95% confidence; **PCC %Change:**change of partial correlation coefficient in percentage compared to the reference model (100%, cursive bold text). **KL:** Kellgren-Lawrence grade (divided into three categories 1, 2 and 3+4 combined), **WC:** Waist Circumference; **SF:** synovial fluid.

**Table S6.** Association between Lequesne and all the parameters evaluated in a **Multivariate model**: effects are simultaneously estimated using a single model including previous confounders and all adipokines except omentin (due to a high collinearity observed with adiponectin, PCC=0.792).

|  | Groups | Adj.Means / Part.Pearson Corr. [95%CI] | Coef. [95%CI] | p-value |
| --- | --- | --- | --- | --- |
| KOA symptoms duration (months) |  | -0.017 [-0.243, 0.211] | -0.093 [-1.368, 1.183] | 0.8852 |
| [Time from radiology](https://880a5856d6b5ee67377e39d185a251f3c007c1f7.googledrive.com/secure/ABCTMEhJZFFKZNgTRAKjNLWqm3r47N9N-bGoHFUT4huGibC4engcdmFUX9Btt0Uqr9K8sXoZGMCZgNxNRmpsRu-pWQOXb_2fwi6Oa9l2VBnnO1y68ziXE6ayMbWty32q8mYyK_p3z7GfaUbupDprQhdDClRzqNODBjEtqbgRKDVvNz7ciwfUjiS9bKRsLTzFWrtkuYB1iI7_41xx2B5rJl0UFLAFsG99ZUX1Uba0D9LsfBq6U26sUR7Ln726HK6dH-xyVzodVG3M9hnbvX_G21aWZttCl58kc37YDvKFpNTSGVt3B1NlPf8oiX9UnAM5KatJde6E1b2XeMcIValBSMTPeToX7YVskG5d92Lh0v5SKU2ZmzKtZUEsYUUYfZDjMjFX0MvldDUokscTaB4v4EFO4tVyndSKMBYT4q92GGw70bl_fwKTeOWUiMFEs6Ki1i-EytW4Bk2ytE-l3S__PrgUhfNJCe3Fldw5bQSsm7YMa8adk-EKazkprHEyb3LTazCKIf8CWohDqGQTEkDC34IKcicqtyKY68dk997n2SNoYRCCxecZTXi-efXkgRB2uPvy3anov0QL/host/0B_MoA5wjzqo7THhvV0hoTlRsaEk/7.11.Pain_multivariant_step_perim_imc_greix_mets_excercici.fisic_omentine_adiponectine/lequesne.t/Basal_Antropometriques.Metaboliques_Inflamatories_Adipoquines.totes.adipoquine.version/Plots/lequesne_radio.months.html) (months) |  | 0.057 [-0.172, 0.281] | 0.019 [-0.056, 0.093] | 0.6159 |
| [Age](https://880a5856d6b5ee67377e39d185a251f3c007c1f7.googledrive.com/secure/ABCTMEhJZFFKZNgTRAKjNLWqm3r47N9N-bGoHFUT4huGibC4engcdmFUX9Btt0Uqr9K8sXoZGMCZgNxNRmpsRu-pWQOXb_2fwi6Oa9l2VBnnO1y68ziXE6ayMbWty32q8mYyK_p3z7GfaUbupDprQhdDClRzqNODBjEtqbgRKDVvNz7ciwfUjiS9bKRsLTzFWrtkuYB1iI7_41xx2B5rJl0UFLAFsG99ZUX1Uba0D9LsfBq6U26sUR7Ln726HK6dH-xyVzodVG3M9hnbvX_G21aWZttCl58kc37YDvKFpNTSGVt3B1NlPf8oiX9UnAM5KatJde6E1b2XeMcIValBSMTPeToX7YVskG5d92Lh0v5SKU2ZmzKtZUEsYUUYfZDjMjFX0MvldDUokscTaB4v4EFO4tVyndSKMBYT4q92GGw70bl_fwKTeOWUiMFEs6Ki1i-EytW4Bk2ytE-l3S__PrgUhfNJCe3Fldw5bQSsm7YMa8adk-EKazkprHEyb3LTazCKIf8CWohDqGQTEkDC34IKcicqtyKY68dk997n2SNoYRCCxecZTXi-efXkgRB2uPvy3anov0QL/host/0B_MoA5wjzqo7THhvV0hoTlRsaEk/7.11.Pain_multivariant_step_perim_imc_greix_mets_excercici.fisic_omentine_adiponectine/lequesne.t/Basal_Antropometriques.Metaboliques_Inflamatories_Adipoquines.totes.adipoquine.version/Plots/lequesne_edat.html) |  | 0.017 [-0.211, 0.243] | 0.007 [-0.085, 0.099] | 0.8836 |
| [KL](https://880a5856d6b5ee67377e39d185a251f3c007c1f7.googledrive.com/secure/ABCTMEhJZFFKZNgTRAKjNLWqm3r47N9N-bGoHFUT4huGibC4engcdmFUX9Btt0Uqr9K8sXoZGMCZgNxNRmpsRu-pWQOXb_2fwi6Oa9l2VBnnO1y68ziXE6ayMbWty32q8mYyK_p3z7GfaUbupDprQhdDClRzqNODBjEtqbgRKDVvNz7ciwfUjiS9bKRsLTzFWrtkuYB1iI7_41xx2B5rJl0UFLAFsG99ZUX1Uba0D9LsfBq6U26sUR7Ln726HK6dH-xyVzodVG3M9hnbvX_G21aWZttCl58kc37YDvKFpNTSGVt3B1NlPf8oiX9UnAM5KatJde6E1b2XeMcIValBSMTPeToX7YVskG5d92Lh0v5SKU2ZmzKtZUEsYUUYfZDjMjFX0MvldDUokscTaB4v4EFO4tVyndSKMBYT4q92GGw70bl_fwKTeOWUiMFEs6Ki1i-EytW4Bk2ytE-l3S__PrgUhfNJCe3Fldw5bQSsm7YMa8adk-EKazkprHEyb3LTazCKIf8CWohDqGQTEkDC34IKcicqtyKY68dk997n2SNoYRCCxecZTXi-efXkgRB2uPvy3anov0QL/host/0B_MoA5wjzqo7THhvV0hoTlRsaEk/7.11.Pain_multivariant_step_perim_imc_greix_mets_excercici.fisic_omentine_adiponectine/lequesne.t/Basal_Antropometriques.Metaboliques_Inflamatories_Adipoquines.totes.adipoquine.version/Plots/lequesne_klb.o3.html) | 1 | 12.308 [10.953, 13.664] | 1.043 [0.125, 1.960] | 0.0265 |
|  | 2 | 13.351 [12.679, 14.022] |  |  |
|  | 3 | 14.393 [13.528, 15.259] |  |  |
| [WC (cm)](https://880a5856d6b5ee67377e39d185a251f3c007c1f7.googledrive.com/secure/ABCTMEhJZFFKZNgTRAKjNLWqm3r47N9N-bGoHFUT4huGibC4engcdmFUX9Btt0Uqr9K8sXoZGMCZgNxNRmpsRu-pWQOXb_2fwi6Oa9l2VBnnO1y68ziXE6ayMbWty32q8mYyK_p3z7GfaUbupDprQhdDClRzqNODBjEtqbgRKDVvNz7ciwfUjiS9bKRsLTzFWrtkuYB1iI7_41xx2B5rJl0UFLAFsG99ZUX1Uba0D9LsfBq6U26sUR7Ln726HK6dH-xyVzodVG3M9hnbvX_G21aWZttCl58kc37YDvKFpNTSGVt3B1NlPf8oiX9UnAM5KatJde6E1b2XeMcIValBSMTPeToX7YVskG5d92Lh0v5SKU2ZmzKtZUEsYUUYfZDjMjFX0MvldDUokscTaB4v4EFO4tVyndSKMBYT4q92GGw70bl_fwKTeOWUiMFEs6Ki1i-EytW4Bk2ytE-l3S__PrgUhfNJCe3Fldw5bQSsm7YMa8adk-EKazkprHEyb3LTazCKIf8CWohDqGQTEkDC34IKcicqtyKY68dk997n2SNoYRCCxecZTXi-efXkgRB2uPvy3anov0QL/host/0B_MoA5wjzqo7THhvV0hoTlRsaEk/7.11.Pain_multivariant_step_perim_imc_greix_mets_excercici.fisic_omentine_adiponectine/lequesne.t/Basal_Antropometriques.Metaboliques_Inflamatories_Adipoquines.totes.adipoquine.version/Plots/lequesne_perimetre.cintura.cm..html) |  | 0.230 [0.003, 0.434] | 0.106 [0.004, 0.209] | 0.0419 |
| %Body fat |  | -0.082 [-0.304, 0.147] | -0.080 [-0.298, 0.139] | 0.4701 |
| [Physical](https://880a5856d6b5ee67377e39d185a251f3c007c1f7.googledrive.com/secure/ABCTMEhJZFFKZNgTRAKjNLWqm3r47N9N-bGoHFUT4huGibC4engcdmFUX9Btt0Uqr9K8sXoZGMCZgNxNRmpsRu-pWQOXb_2fwi6Oa9l2VBnnO1y68ziXE6ayMbWty32q8mYyK_p3z7GfaUbupDprQhdDClRzqNODBjEtqbgRKDVvNz7ciwfUjiS9bKRsLTzFWrtkuYB1iI7_41xx2B5rJl0UFLAFsG99ZUX1Uba0D9LsfBq6U26sUR7Ln726HK6dH-xyVzodVG3M9hnbvX_G21aWZttCl58kc37YDvKFpNTSGVt3B1NlPf8oiX9UnAM5KatJde6E1b2XeMcIValBSMTPeToX7YVskG5d92Lh0v5SKU2ZmzKtZUEsYUUYfZDjMjFX0MvldDUokscTaB4v4EFO4tVyndSKMBYT4q92GGw70bl_fwKTeOWUiMFEs6Ki1i-EytW4Bk2ytE-l3S__PrgUhfNJCe3Fldw5bQSsm7YMa8adk-EKazkprHEyb3LTazCKIf8CWohDqGQTEkDC34IKcicqtyKY68dk997n2SNoYRCCxecZTXi-efXkgRB2uPvy3anov0QL/host/0B_MoA5wjzqo7THhvV0hoTlRsaEk/7.11.Pain_multivariant_step_perim_imc_greix_mets_excercici.fisic_omentine_adiponectine/lequesne.t/Basal_Antropometriques.Metaboliques_Inflamatories_Adipoquines.totes.adipoquine.version/Plots/lequesne_exercici.fisic.c3.html) exercise | Never | 14.693 [13.710, 15.676] | Ref. | 0.0016 |
|  | Occasional | 14.039 [12.698, 15.381] | -0.653 [-2.435, 1.128] |  |
|  | Regular | 11.151 [9.671, 12.630] | -3.542 [-5.522, -1.562] |  |
| TNF-alpha SF |  | 0.131 [-0.099, 0.348] | 1.240 [-0.889, 3.368] | 0.2497 |
| Leptin SF |  | 0.192 [-0.036, 0.402] | 0.291 [-0.046, 0.628] | 0.0897 |
| Adiponectin SF |  | 0.212 [-0.016, 0.419] | 99.235 [-4.756, 203.226] | 0.0612 |
| Resistin SF |  | 0.265 [0.040, 0.464] | 31.308 [5.424, 57.191] | 0.0184 |
| Visfatin SF |  | -0.298 [-0.492, -0.077] | -1.205 [-2.079, -0.331] | 0.0075 |
| [Osteopontin](https://880a5856d6b5ee67377e39d185a251f3c007c1f7.googledrive.com/secure/ABCTMEhJZFFKZNgTRAKjNLWqm3r47N9N-bGoHFUT4huGibC4engcdmFUX9Btt0Uqr9K8sXoZGMCZgNxNRmpsRu-pWQOXb_2fwi6Oa9l2VBnnO1y68ziXE6ayMbWty32q8mYyK_p3z7GfaUbupDprQhdDClRzqNODBjEtqbgRKDVvNz7ciwfUjiS9bKRsLTzFWrtkuYB1iI7_41xx2B5rJl0UFLAFsG99ZUX1Uba0D9LsfBq6U26sUR7Ln726HK6dH-xyVzodVG3M9hnbvX_G21aWZttCl58kc37YDvKFpNTSGVt3B1NlPf8oiX9UnAM5KatJde6E1b2XeMcIValBSMTPeToX7YVskG5d92Lh0v5SKU2ZmzKtZUEsYUUYfZDjMjFX0MvldDUokscTaB4v4EFO4tVyndSKMBYT4q92GGw70bl_fwKTeOWUiMFEs6Ki1i-EytW4Bk2ytE-l3S__PrgUhfNJCe3Fldw5bQSsm7YMa8adk-EKazkprHEyb3LTazCKIf8CWohDqGQTEkDC34IKcicqtyKY68dk997n2SNoYRCCxecZTXi-efXkgRB2uPvy3anov0QL/host/0B_MoA5wjzqo7THhvV0hoTlRsaEk/7.11.Pain_multivariant_step_perim_imc_greix_mets_excercici.fisic_omentine_adiponectine/lequesne.t/Basal_Antropometriques.Metaboliques_Inflamatories_Adipoquines.totes.adipoquine.version/Plots/lequesne_osteopontina.ls.ngr.ml.html) SF |  | 0.197 [-0.032, 0.406] | 0.672 [-0.088, 1.431] | 0.0824 |
| [Chemerin](https://880a5856d6b5ee67377e39d185a251f3c007c1f7.googledrive.com/secure/ABCTMEhJZFFKZNgTRAKjNLWqm3r47N9N-bGoHFUT4huGibC4engcdmFUX9Btt0Uqr9K8sXoZGMCZgNxNRmpsRu-pWQOXb_2fwi6Oa9l2VBnnO1y68ziXE6ayMbWty32q8mYyK_p3z7GfaUbupDprQhdDClRzqNODBjEtqbgRKDVvNz7ciwfUjiS9bKRsLTzFWrtkuYB1iI7_41xx2B5rJl0UFLAFsG99ZUX1Uba0D9LsfBq6U26sUR7Ln726HK6dH-xyVzodVG3M9hnbvX_G21aWZttCl58kc37YDvKFpNTSGVt3B1NlPf8oiX9UnAM5KatJde6E1b2XeMcIValBSMTPeToX7YVskG5d92Lh0v5SKU2ZmzKtZUEsYUUYfZDjMjFX0MvldDUokscTaB4v4EFO4tVyndSKMBYT4q92GGw70bl_fwKTeOWUiMFEs6Ki1i-EytW4Bk2ytE-l3S__PrgUhfNJCe3Fldw5bQSsm7YMa8adk-EKazkprHEyb3LTazCKIf8CWohDqGQTEkDC34IKcicqtyKY68dk997n2SNoYRCCxecZTXi-efXkgRB2uPvy3anov0QL/host/0B_MoA5wjzqo7THhvV0hoTlRsaEk/7.11.Pain_multivariant_step_perim_imc_greix_mets_excercici.fisic_omentine_adiponectine/lequesne.t/Basal_Antropometriques.Metaboliques_Inflamatories_Adipoquines.totes.adipoquine.version/Plots/lequesne_quemerina.ngr.ml.html) SF |  | 0.012 [-0.215, 0.239] | 1.387 [-23.895, 26.669] | 0.9133 |

**KL:** Kellgren-Lawrence grade (divided into three categories 1, 2 and 3+4 combined) **WC:** Waist Circumference; **SF:** synovial fluid.

**Table S7.** Association between Lequesne and all the parameters evaluated in a **Multivariate model**: effects are simultaneously estimated using a single model including previous confounders and all adipokines except adiponectin (due to a high collinearity observed with omentin, PCC=0.792).

|  | Groups | Adj.Means / Part.Pearson Corr. [95%CI] | Coef. [95%CI] | p-value |
| --- | --- | --- | --- | --- |
| KOA symptoms duration (months) |  | -0.051 [-0.275, 0.178] | -0.290 [-1.569, 0.989] | 0.6528 |
| [Months to radiology](https://880a5856d6b5ee67377e39d185a251f3c007c1f7.googledrive.com/secure/ABCTMEhJZFFKZNgTRAKjNLWqm3r47N9N-bGoHFUT4huGibC4engcdmFUX9Btt0Uqr9K8sXoZGMCZgNxNRmpsRu-pWQOXb_2fwi6Oa9l2VBnnO1y68ziXE6ayMbWty32q8mYyK_p3z7GfaUbupDprQhdDClRzqNODBjEtqbgRKDVvNz7ciwfUjiS9bKRsLTzFWrtkuYB1iI7_41xx2B5rJl0UFLAFsG99ZUX1Uba0D9LsfBq6U26sUR7Ln726HK6dH-xyVzodVG3M9hnbvX_G21aWZttCl58kc37YDvKFpNTSGVt3B1NlPf8oiX9UnAM5KatJde6E1b2XeMcIValBSMTPeToX7YVskG5d92Lh0v5SKU2ZmzKtZUEsYUUYfZDjMjFX0MvldDUokscTaB4v4EFO4tVyndSKMBYT4q92GGw70bl_fwKTeOWUiMFEs6Ki1i-EytW4Bk2ytE-l3S__PrgUhfNJCe3Fldw5bQSsm7YMa8adk-EKazkprHEyb3LTazCKIf8CWohDqGQTEkDC34IKcicqtyKY68dk997n2SNoYRCCxecZTXi-efXkgRB2uPvy3anov0QL/host/0B_MoA5wjzqo7THhvV0hoTlRsaEk/7.11.Pain_multivariant_step_perim_imc_greix_mets_excercici.fisic_omentine_adiponectine/lequesne.t/Basal_Antropometriques.Metaboliques_Inflamatories_Adipoquines.totes.adipoquine.version/Plots/lequesne_radio.months.html) |  | 0.070 [-0.160, 0.292] | 0.023 [-0.052, 0.097] | 0.5425 |
| [Age](https://880a5856d6b5ee67377e39d185a251f3c007c1f7.googledrive.com/secure/ABCTMEhJZFFKZNgTRAKjNLWqm3r47N9N-bGoHFUT4huGibC4engcdmFUX9Btt0Uqr9K8sXoZGMCZgNxNRmpsRu-pWQOXb_2fwi6Oa9l2VBnnO1y68ziXE6ayMbWty32q8mYyK_p3z7GfaUbupDprQhdDClRzqNODBjEtqbgRKDVvNz7ciwfUjiS9bKRsLTzFWrtkuYB1iI7_41xx2B5rJl0UFLAFsG99ZUX1Uba0D9LsfBq6U26sUR7Ln726HK6dH-xyVzodVG3M9hnbvX_G21aWZttCl58kc37YDvKFpNTSGVt3B1NlPf8oiX9UnAM5KatJde6E1b2XeMcIValBSMTPeToX7YVskG5d92Lh0v5SKU2ZmzKtZUEsYUUYfZDjMjFX0MvldDUokscTaB4v4EFO4tVyndSKMBYT4q92GGw70bl_fwKTeOWUiMFEs6Ki1i-EytW4Bk2ytE-l3S__PrgUhfNJCe3Fldw5bQSsm7YMa8adk-EKazkprHEyb3LTazCKIf8CWohDqGQTEkDC34IKcicqtyKY68dk997n2SNoYRCCxecZTXi-efXkgRB2uPvy3anov0QL/host/0B_MoA5wjzqo7THhvV0hoTlRsaEk/7.11.Pain_multivariant_step_perim_imc_greix_mets_excercici.fisic_omentine_adiponectine/lequesne.t/Basal_Antropometriques.Metaboliques_Inflamatories_Adipoquines.totes.adipoquine.version/Plots/lequesne_edat.html) |  | 0.034 [-0.194, 0.259] | 0.014 [-0.079, 0.107] | 0.7662 |
| [KL](https://880a5856d6b5ee67377e39d185a251f3c007c1f7.googledrive.com/secure/ABCTMEhJZFFKZNgTRAKjNLWqm3r47N9N-bGoHFUT4huGibC4engcdmFUX9Btt0Uqr9K8sXoZGMCZgNxNRmpsRu-pWQOXb_2fwi6Oa9l2VBnnO1y68ziXE6ayMbWty32q8mYyK_p3z7GfaUbupDprQhdDClRzqNODBjEtqbgRKDVvNz7ciwfUjiS9bKRsLTzFWrtkuYB1iI7_41xx2B5rJl0UFLAFsG99ZUX1Uba0D9LsfBq6U26sUR7Ln726HK6dH-xyVzodVG3M9hnbvX_G21aWZttCl58kc37YDvKFpNTSGVt3B1NlPf8oiX9UnAM5KatJde6E1b2XeMcIValBSMTPeToX7YVskG5d92Lh0v5SKU2ZmzKtZUEsYUUYfZDjMjFX0MvldDUokscTaB4v4EFO4tVyndSKMBYT4q92GGw70bl_fwKTeOWUiMFEs6Ki1i-EytW4Bk2ytE-l3S__PrgUhfNJCe3Fldw5bQSsm7YMa8adk-EKazkprHEyb3LTazCKIf8CWohDqGQTEkDC34IKcicqtyKY68dk997n2SNoYRCCxecZTXi-efXkgRB2uPvy3anov0QL/host/0B_MoA5wjzqo7THhvV0hoTlRsaEk/7.11.Pain_multivariant_step_perim_imc_greix_mets_excercici.fisic_omentine_adiponectine/lequesne.t/Basal_Antropometriques.Metaboliques_Inflamatories_Adipoquines.totes.adipoquine.version/Plots/lequesne_klb.o3.html) | 1 | 12.202 [10.856, 13.549] | 1.123 [0.212, 2.033] | 0.0164 |
|  | 2 | 13.325 [12.655, 13.995] |  |  |
|  | 3 | 14.448 [13.586, 15.309] |  |  |
| [WC (cm)](https://880a5856d6b5ee67377e39d185a251f3c007c1f7.googledrive.com/secure/ABCTMEhJZFFKZNgTRAKjNLWqm3r47N9N-bGoHFUT4huGibC4engcdmFUX9Btt0Uqr9K8sXoZGMCZgNxNRmpsRu-pWQOXb_2fwi6Oa9l2VBnnO1y68ziXE6ayMbWty32q8mYyK_p3z7GfaUbupDprQhdDClRzqNODBjEtqbgRKDVvNz7ciwfUjiS9bKRsLTzFWrtkuYB1iI7_41xx2B5rJl0UFLAFsG99ZUX1Uba0D9LsfBq6U26sUR7Ln726HK6dH-xyVzodVG3M9hnbvX_G21aWZttCl58kc37YDvKFpNTSGVt3B1NlPf8oiX9UnAM5KatJde6E1b2XeMcIValBSMTPeToX7YVskG5d92Lh0v5SKU2ZmzKtZUEsYUUYfZDjMjFX0MvldDUokscTaB4v4EFO4tVyndSKMBYT4q92GGw70bl_fwKTeOWUiMFEs6Ki1i-EytW4Bk2ytE-l3S__PrgUhfNJCe3Fldw5bQSsm7YMa8adk-EKazkprHEyb3LTazCKIf8CWohDqGQTEkDC34IKcicqtyKY68dk997n2SNoYRCCxecZTXi-efXkgRB2uPvy3anov0QL/host/0B_MoA5wjzqo7THhvV0hoTlRsaEk/7.11.Pain_multivariant_step_perim_imc_greix_mets_excercici.fisic_omentine_adiponectine/lequesne.t/Basal_Antropometriques.Metaboliques_Inflamatories_Adipoquines.totes.adipoquine.version/Plots/lequesne_perimetre.cintura.cm..html) |  | 0.243 [0.017, 0.446] | 0.116 [0.011, 0.221] | 0.0308 |
| %Body fat |  | -0.092 [-0.312, 0.138] | -0.089 [-0.308, 0.130] | 0.4202 |
| [Physical](https://880a5856d6b5ee67377e39d185a251f3c007c1f7.googledrive.com/secure/ABCTMEhJZFFKZNgTRAKjNLWqm3r47N9N-bGoHFUT4huGibC4engcdmFUX9Btt0Uqr9K8sXoZGMCZgNxNRmpsRu-pWQOXb_2fwi6Oa9l2VBnnO1y68ziXE6ayMbWty32q8mYyK_p3z7GfaUbupDprQhdDClRzqNODBjEtqbgRKDVvNz7ciwfUjiS9bKRsLTzFWrtkuYB1iI7_41xx2B5rJl0UFLAFsG99ZUX1Uba0D9LsfBq6U26sUR7Ln726HK6dH-xyVzodVG3M9hnbvX_G21aWZttCl58kc37YDvKFpNTSGVt3B1NlPf8oiX9UnAM5KatJde6E1b2XeMcIValBSMTPeToX7YVskG5d92Lh0v5SKU2ZmzKtZUEsYUUYfZDjMjFX0MvldDUokscTaB4v4EFO4tVyndSKMBYT4q92GGw70bl_fwKTeOWUiMFEs6Ki1i-EytW4Bk2ytE-l3S__PrgUhfNJCe3Fldw5bQSsm7YMa8adk-EKazkprHEyb3LTazCKIf8CWohDqGQTEkDC34IKcicqtyKY68dk997n2SNoYRCCxecZTXi-efXkgRB2uPvy3anov0QL/host/0B_MoA5wjzqo7THhvV0hoTlRsaEk/7.11.Pain_multivariant_step_perim_imc_greix_mets_excercici.fisic_omentine_adiponectine/lequesne.t/Basal_Antropometriques.Metaboliques_Inflamatories_Adipoquines.totes.adipoquine.version/Plots/lequesne_exercici.fisic.c3.html) exercise | Never | 14.614 [13.634, 15.594] | Ref. | 0.0020 |
|  | Occasional | 14.150 [12.803, 15.497] | -0.464 [-2.250, 1.322] |  |
|  | Regular | 11.212 [9.739, 12.684] | -3.402 [-5.369, -1.436] |  |
| TNF-alpha SF |  | 0.152 [-0.077, 0.367] | 1.446 [-0.681, 3.574] | 0.1798 |
| Leptin SF |  | 0.189 [-0.040, 0.399] | 0.286 [-0.051, 0.623] | 0.0956 |
| Resistin SF |  | 0.274 [0.050, 0.471] | 32.752 [6.635, 58.869] | 0.0147 |
| Visfatin SF |  | -0.298 [-0.492, -0.076] | -1.195 [-2.064, -0.326] | 0.0077 |
| [Osteopontin](https://880a5856d6b5ee67377e39d185a251f3c007c1f7.googledrive.com/secure/ABCTMEhJZFFKZNgTRAKjNLWqm3r47N9N-bGoHFUT4huGibC4engcdmFUX9Btt0Uqr9K8sXoZGMCZgNxNRmpsRu-pWQOXb_2fwi6Oa9l2VBnnO1y68ziXE6ayMbWty32q8mYyK_p3z7GfaUbupDprQhdDClRzqNODBjEtqbgRKDVvNz7ciwfUjiS9bKRsLTzFWrtkuYB1iI7_41xx2B5rJl0UFLAFsG99ZUX1Uba0D9LsfBq6U26sUR7Ln726HK6dH-xyVzodVG3M9hnbvX_G21aWZttCl58kc37YDvKFpNTSGVt3B1NlPf8oiX9UnAM5KatJde6E1b2XeMcIValBSMTPeToX7YVskG5d92Lh0v5SKU2ZmzKtZUEsYUUYfZDjMjFX0MvldDUokscTaB4v4EFO4tVyndSKMBYT4q92GGw70bl_fwKTeOWUiMFEs6Ki1i-EytW4Bk2ytE-l3S__PrgUhfNJCe3Fldw5bQSsm7YMa8adk-EKazkprHEyb3LTazCKIf8CWohDqGQTEkDC34IKcicqtyKY68dk997n2SNoYRCCxecZTXi-efXkgRB2uPvy3anov0QL/host/0B_MoA5wjzqo7THhvV0hoTlRsaEk/7.11.Pain_multivariant_step_perim_imc_greix_mets_excercici.fisic_omentine_adiponectine/lequesne.t/Basal_Antropometriques.Metaboliques_Inflamatories_Adipoquines.totes.adipoquine.version/Plots/lequesne_osteopontina.ls.ngr.ml.html) SF |  | 0.182 [-0.046, 0.393] | 0.631 [-0.141, 1.402] | 0.1076 |
| Omentin SF |  | 0.216 [-0.012, 0.422] | 0.854 [-0.023, 1.731] | 0.0562 |
| [Chemerin](https://880a5856d6b5ee67377e39d185a251f3c007c1f7.googledrive.com/secure/ABCTMEhJZFFKZNgTRAKjNLWqm3r47N9N-bGoHFUT4huGibC4engcdmFUX9Btt0Uqr9K8sXoZGMCZgNxNRmpsRu-pWQOXb_2fwi6Oa9l2VBnnO1y68ziXE6ayMbWty32q8mYyK_p3z7GfaUbupDprQhdDClRzqNODBjEtqbgRKDVvNz7ciwfUjiS9bKRsLTzFWrtkuYB1iI7_41xx2B5rJl0UFLAFsG99ZUX1Uba0D9LsfBq6U26sUR7Ln726HK6dH-xyVzodVG3M9hnbvX_G21aWZttCl58kc37YDvKFpNTSGVt3B1NlPf8oiX9UnAM5KatJde6E1b2XeMcIValBSMTPeToX7YVskG5d92Lh0v5SKU2ZmzKtZUEsYUUYfZDjMjFX0MvldDUokscTaB4v4EFO4tVyndSKMBYT4q92GGw70bl_fwKTeOWUiMFEs6Ki1i-EytW4Bk2ytE-l3S__PrgUhfNJCe3Fldw5bQSsm7YMa8adk-EKazkprHEyb3LTazCKIf8CWohDqGQTEkDC34IKcicqtyKY68dk997n2SNoYRCCxecZTXi-efXkgRB2uPvy3anov0QL/host/0B_MoA5wjzqo7THhvV0hoTlRsaEk/7.11.Pain_multivariant_step_perim_imc_greix_mets_excercici.fisic_omentine_adiponectine/lequesne.t/Basal_Antropometriques.Metaboliques_Inflamatories_Adipoquines.totes.adipoquine.version/Plots/lequesne_quemerina.ngr.ml.html) SF |  | -0.005 [-0.232, 0.222] | -0.582 [-25.800, 24.635] | 0.9634 |

**KL:** Kellgren-Lawrence grade (divided into three categories 1, 2 and 3+4 combined) **WC:** Waist Circumference; **SF:** synovial fluid.

|  | Groups | Adj.Means / Part.Pearson Corr.  [95%CI] | Coef.  [95%CI] | F-pval |
| --- | --- | --- | --- | --- |
| KOA symptoms duration |  | -0.075  [-0.294, 0.152] | -0.424  [-1.691, 0.842] | 0.5067 |
| [Time](http://www.aberenguer.com/JCalvet_201508/15.Pain_multivariant_step_referee_suggestion_no_anthrop_metabol/lequesne.t/Adipoquines.all,.adiponectine.version_basal.selected_TNF.alfa.forced/Plots/lequesne_radio.months.html) from radiology (months) |  | 0.008  [-0.216, 0.232] | 0.003  [-0.072, 0.077] | 0.9428 |
| [Age](http://www.aberenguer.com/JCalvet_201508/15.Pain_multivariant_step_referee_suggestion_no_anthrop_metabol/lequesne.t/Adipoquines.all,.adiponectine.version_basal.selected_TNF.alfa.forced/Plots/lequesne_edat.html) |  | 0.013  [-0.212, 0.236] | 0.005  [-0.089, 0.099] | 0.9105 |
| [KL](http://www.aberenguer.com/JCalvet_201508/15.Pain_multivariant_step_referee_suggestion_no_anthrop_metabol/lequesne.t/Adipoquines.all,.adiponectine.version_basal.selected_TNF.alfa.forced/Plots/lequesne_klb.o3.html) | 1 | 12.262  [10.880, 13.645] | 1.077  [0.142, 2.013] | 0.0246 |
|  | 2 | 13.340  [12.654, 14.025] |  |  |
|  | 3 | 14.417  [13.534, 15.300] |  |  |
| Physical exercise | Never | 14.888  [13.900, 15.877] | Ref. | 0.000945 |
|  | Occasional | 13.751  [12.405, 15.096] | -1.138  [-2.905, 0.630] |  |
|  | Regular | 11.014  [9.509, 12.519] | -3.874  [-5.874, -1.875] |  |
| Leptin SF |  | 0.300  [0.081, 0.491] | 0.431  [0.124, 0.738] | 0.00655 |
| Adiponectin SF |  | 0.140  [-0.087, 0.353] | 64.078  [-37.296, 165.453] | 0.2120 |
| Resistin SF |  | 0.259  [0.037, 0.457] | 31.464  [5.219, 57.708] | 0.0194 |
| Visfatin SF |  | -0.264  [-0.461, -0.042] | -1.077  [-1.959, -0.196] | 0.0173 |
| Osteopontin SF |  | 0.281  [0.061, 0.475] | 0.955  [0.225, 1.685] | 0.0110 |
| Chemerin SF |  | -0.026  [-0.249, 0.199] | -2.988  [-28.494, 22.517] | 0.8162 |
| TNF-alpha SF |  | 0.143  [-0.084, 0.355] | 1.393  [-0.768, 3.555] | 0.2032 |

**Table S8**. Association between Lequesne and all parameters evaluated in a **Multivariate model**: effects are simultaneosly estimated using a single model including previous confounders except anthropometric and metabolic parameters and all adipokines except omentin (due to high collinearity observed with adiponectin, PCC=0.792).

**KL:** Kellgren-Lawrence grade (divided into three categories 1, 2 and 3+4 combined) **WC:** Waist Circumference; **SF:** synovial fluid.

**Table S9.** Association between Lequesne and all parameters evaluated in a **Multivariate model**: effects are simultaneosly estimated using a single model including previous confounders except inflammatory markers and all adipokines except omentin (due to high collinearity observed with adiponectin, PCC=0.792).

|  | Groups | Adj.Means / Part.Pearson Corr.  [95%CI] | Coef.  [95%CI] | F-pval |
| --- | --- | --- | --- | --- |
| KOA symptoms duration (months) |  | -0.026  [-0.236, 0.187] | -0.151  [-1.387, 1.085] | 0.8090 |
| [Time](http://www.aberenguer.com/JCalvet_201508/16.Sequential_fits_adding_notinflamm_models/lequesne.t/Basal_Antropometriques.Metaboliques_.Adipoquines.totes.adipoquine.version/Plots/lequesne_radio.months.html) from radiology (months) |  | 0.085  [-0.129, 0.291] | 0.029  [-0.043, 0.101] | 0.4268 |
| [Age](http://www.aberenguer.com/JCalvet_201508/16.Sequential_fits_adding_notinflamm_models/lequesne.t/Basal_Antropometriques.Metaboliques_.Adipoquines.totes.adipoquine.version/Plots/lequesne_edat.html) |  | -0.040  [-0.250, 0.174] | -0.017  [-0.110, 0.075] | 0.7098 |
| [KL](http://www.aberenguer.com/JCalvet_201508/16.Sequential_fits_adding_notinflamm_models/lequesne.t/Basal_Antropometriques.Metaboliques_.Adipoquines.totes.adipoquine.version/Plots/lequesne_klb.o3.html) | 1 | 12.179  [10.830, 13.528] | 1.185  [0.276, 2.093] | 0.0112 |
|  | 2 | 13.364  [12.692, 14.035] |  |  |
|  | 3 | 14.548  [13.693, 15.403] |  |  |
| [Physical](http://www.aberenguer.com/JCalvet_201508/16.Sequential_fits_adding_notinflamm_models/lequesne.t/Basal_Antropometriques.Metaboliques_.Adipoquines.totes.adipoquine.version/Plots/lequesne_exercici.fisic.c3.html) exercise | Cap | 14.696  [13.641, 15.751] | Ref. | 0.0315 |
|  | Esporadic | 13.821  [12.501, 15.140] | -0.875  [-2.685, 0.935] |  |
|  | Regular | 12.129  [10.775, 13.484] | -2.566  [-4.501, -0.631] |  |
| [WC](http://www.aberenguer.com/JCalvet_201508/16.Sequential_fits_adding_notinflamm_models/lequesne.t/Basal_Antropometriques.Metaboliques_.Adipoquines.totes.adipoquine.version/Plots/lequesne_perimetre.cintura.cm..html) (cm) |  | 0.228  [0.017, 0.419] | 0.113  [0.011, 0.215] | 0.0310 |
| [%](http://www.aberenguer.com/JCalvet_201508/16.Sequential_fits_adding_notinflamm_models/lequesne.t/Basal_Antropometriques.Metaboliques_.Adipoquines.totes.adipoquine.version/Plots/lequesne_x.greix.html) Body Fat |  | -0.109  [-0.314, 0.105] | -0.109  [-0.319, 0.101] | 0.3058 |
| Leptin SF |  | 0.234  [0.023, 0.425] | 0.373  [0.045, 0.700] | 0.0264 |
| [Adiponectin](http://www.aberenguer.com/JCalvet_201508/16.Sequential_fits_adding_notinflamm_models/lequesne.t/Basal_Antropometriques.Metaboliques_.Adipoquines.totes.adipoquine.version/Plots/lequesne_adiponectina.ls.ng.ml.html) SF |  | 0.152  [-0.062, 0.352] | 71.180  [-26.953, 169.313] | 0.1530 |
| Resistin SF |  | 0.251  [0.041, 0.439] | 29.858  [5.417, 54.298] | 0.0172 |
| Visfatin SF |  | -0.232  [-0.423, -0.021] | -0.968  [-1.830, -0.106] | 0.0281 |
| Osteopontin SF |  | 0.204  [-0.008, 0.399] | 0.671  [-0.011, 1.353] | 0.0536 |
| Chemerin SF |  | -0.004  [-0.216, 0.208] | -0.497  [-25.548, 24.555] | 0.9687 |

**KL:** Kellgren-Lawrence grade (divided into three categories 1, 2 and 3+4 combined) **WC:** Waist Circumference; **SF:** synovial fluid.

**Table S10**. Different adipokine levels separatelly by KL degree.

|  | KL 1  Median (IQR) | KL 2  Median (IQR) | KL 3  Median (IQR) | KL 4  Median (IQR) | P value |
| --- | --- | --- | --- | --- | --- |
| [Leptin](http://www.aberenguer.com/JCalvet_201508/3.Assoc_univ_females/leptina.ls.pgr.ml_klb/Plots/leptina.ls.pgr.ml_klb.html) SF | 43.340.718  [32741.275, 56326.580] | 44.423.960  [38423.583, 51101.112] | 45.527.382  [38425.318, 53571.224] | 46.651.233  [33971.943, 62586.682] | 0.7782 |
| [Adiponectin](http://www.aberenguer.com/JCalvet_201508/3.Assoc_univ_females/adiponectina.ls.ng.ml_klb/Plots/adiponectina.ls.ng.ml_klb.html) SF | 1.521.006  [1239.907, 1909.774] | 1.617.137  [1443.153, 1824.591] | 1.722.679  [1498.021, 2001.942] | 1.838.901  [1419.808, 2475.152] | 0.3934 |
| [Resistin](http://www.aberenguer.com/JCalvet_201508/3.Assoc_univ_females/resistina.ls.pgr.ml_klb/Plots/resistina.ls.pgr.ml_klb.html) SF | 2.387.091  [1693.809, 3474.270] | 2.114.931  [1767.550, 2552.001] | 1.880.476  [1527.562, 2341.453] | 1.677.627  [1163.873, 2508.844] | 0.2970 |
| [Visfatin](http://www.aberenguer.com/JCalvet_201508/3.Assoc_univ_females/visfatina.ls.ngr.ml_klb/Plots/visfatina.ls.ngr.ml_klb.html) SF | 1.398  [0.992, 1.970] | 1.565  [1.306, 1.876] | 1.753  [1.411, 2.178] | 1.963  [1.316, 2.930] | 0.3154 |
| [Osteopontin](http://www.aberenguer.com/JCalvet_201508/3.Assoc_univ_females/osteopontina.ls.ngr.ml_klb/Plots/osteopontina.ls.ngr.ml_klb.html) SF | 66.951  [43.077, 104.056] | 69.902  [55.382, 88.228] | 72.982  [55.228, 96.445] | 76.199  [45.542, 127.493] | 0.7657 |
| [Omentin](http://www.aberenguer.com/JCalvet_201508/3.Assoc_univ_females/omentina.pgr.ml_klb/Plots/omentina.pgr.ml_klb.html) SF | 3.042.336  [2195.958, 4214.929] | 3.248.367  [2737.708, 3854.277] | 3.468.350  [2825.035, 4258.159] | 3.703.230  [2531.181, 5417.991] | 0.5413 |
| [Chemerin](http://www.aberenguer.com/JCalvet_201508/3.Assoc_univ_females/quemerina.ngr.ml_klb/Plots/quemerina.ngr.ml_klb.html) SF | 89.168  [72.571, 112.184] | 98.493  [87.577, 111.585] | 109.362  [94.347, 128.269] | 122.134  [92.524, 168.617] | 0.1750 |
